# N-Acetylcysteine Improves Liver Function in Patients with Non-Alcoholic Fatty Liver Disease

**Published:** 2010-03-01

**Authors:** Manouchehr Khoshbaten, Akbar Aliasgarzadeh, Koorosh Masnadi, Mohammad K Tarzamani, Sara Farhang, Hosain Babaei, Javad Kiani, Maryam Zaare, Farzad Najafipoor

**Affiliations:** 1 Drug Applied Research Center, Tabriz University of Medical Sciences, Tabriz, Iran; 2 Department of Endocrinology, Tabriz University of Medical Sciences, Tabriz, Iran; 3 Department of Radiology, Tabriz University of Medical Sciences, Tabriz, Iran; 4 Liver and Gastrointestinal Diseases Research Center, Imam Reza Hospital, Tabriz, Iran; 5 Department of Pharmacology, Tabriz University of Medical Sciences, Tabriz, Iran; 6 Department of Internal Medicine, Tabriz University of Medical Sciences, Tabriz, Iran

**Keywords:** Non-alcoholic Fatty Liver Disease, N-acetylcysteine, Therapy

## Abstract

**Background and Aims:**

Non-alcoholic fatty liver change is a common disease of the liver in which oxidative stress plays a basic role. Studies are largely focused on protecting the liver by means of anti-oxidative material. The aim of this study is to evaluate the role of N- acetylcysteine in the process of liver injury.

**Methods:**

Thirty patients with non-alcoholic fatty liver steatosis were randomly selected to receive either N-acetylcysteine or vitamin C. Liver function tests (alanine aminotransfrase, aspartate aminotransfrase and alkaline phosphatase) were measured as well as the grade of steatosis, the pattern of its echogenicity, the span of the liver and the spleen and the portal vein diameter before the intervention. Patients were followed up using the same method of evaluation repeated in the first, second and third months.

**Results:**

The mean age (SD) was 40.1(12.4) in patients receiving NAC and 46(10.4) years in patients receiving vitamin C (P = 0.137). NAC resulted in a significant decrease of serum alanine aminotransfrase after three months, compared to vitamin C. This effect was independent of the grade of steatosis in the initial diagnosis. NAC was able to significantly decrease the span of the spleen.

**Conclusions:**

N-acetylcysteine can improve liver function in patients with non-alcoholic fatty liver disease. Better results may be achievable in a longer follow up.

## Introduction

Non-alcoholic fatty liver disease (NAFLD) is very common and affects up to 24 % of the general population in various countries [1].An increased level of fatty acids within the hepatocytes causing oxidative stress, is believed to be responsible for the progression from steatosis to steatohepatitis and cirrhosis. The elevated serum levels of liver enzymes may be the only laboratory clues during this progress [2].

Studies focus on anti-oxidative material in order to find protection against liver injury. Vitamins C and E have been found to have both protective and therapeutic effects on NAFLD in an animal model [3][4]. This effect has been linked to the anti-oxidative function of these two vitamins. Nevertheless no medication ha been definitively established for NAFLD and research has tried different free-radical scavengers and antioxidant agents.

N-acetylcysteine (NAC) is frequently utilized where intracellular oxidant-antioxidant balance isconcerned. NAC has a protective effect against liver injury in rats [5][8]. One study has reported improvement of liver histopathology and reduction of oxidative stress by NAC in non-alcoholic fatty steatosis (NASH) in a rat model [9]. A recent study has reported a significant decrease in liver steatosis and fibrosis in patients with NASH receiving Metformin and NAC [10]. In this study, the significance of treatment with NAC in patients with NAFLD and with elevated liver enzymes has been investigated.

## Materials and methods

This study was carried out in the clinics of Tabriz University of Medical Sciences during 2008 and the protocols were approved by the Ethical Committee of this University.

Patients with ongoing NAFLD were included in this study and received either NAC (600 mg per 12 hours) or vitamin C (1000 mg per 12 hours).Patients with a diagnosis of NAFLD are commonly registered in this clinic. Patients would pick up a ticket from a box containing mixed labels of two different treatments, twenty from each category.Data collection was stopped when fifteen patients completed the treatment and the follow-up

Most of the patients were referred because of elevated liver enzymes or a report of fatty liver in sonographic evaluation and some had nonspecific complaints like abdominal discomfort. All patients gave their written consent. Patients received medication for medical conditions if necessary.

The results of the clinical examination, the elevated level of liver enzymes, an ultrasonographic study of the liver and the exclusion of other etiologies for liver disease were the basis for the diagnosis ofNAFLD. A detailed history was taken and patients with a history of alcohol consumption or the use of medications known to precipitate steatohepatitis, lipid-reducing agents, ursodeoxycholic acid or vitamin supplements in the 6 months prior to study were excluded.

Laboratory evaluation in this study included serum liver tests: aspartate transaminase (AST), alanine transaminase (ALT), and alkaline phosphatase (ALP). Further investigations included a hepatobiliarysystem ultrasound, viral serology, autoantibodytiters, serum iron, ferritin and transferrin saturation, ceruloplasmin and urine copper levels. Serologicaltests for hepatitis B, the antibody to the hepatitis C virus or autoantibodies, i.e., antinuclear antibody (ANA), antimitochondrial antibodies (AMA), anti-smooth muscle antibody (ASMA), and antibodies to liver-kidney microsome (anti-LKM), were negative in all patients.

Serum electrolytes, urea, creatinine, fasting glucose, complete blood count, cholesterol and triglyceride levels were also obtained. An ion profile, a1-antitripsine and serum ceruloplasmin levels were normal in all patients.Serum biochemistry and ultrasonographic measurement of the liver and the spleen were performed at entry and every month for the period of the study. Liver hemodynamics, the grade ofsteatosis and the size of the spleen were measured by the same radiologist, blinded to the treatment method of the patients.

All data were expressed as the mean ± standard deviation (SD). A chi-squared test was used to compare qualitative variables and Student’s t-test was used to compare quantitative variables between the two groups of patients. A repeated-measures ANOVA and k-related-samples tests were used to compare the data before and during each treatment where appropriate. A P-value of 0.05 was considered statistically significant.

## Results

Fifteen patients completed the study procedure and were available for a full follow-up in each group as described above. The study population included 6 males and 9 females treated with NAC and 5 males and 10 females treated with vitamin C. No adverse drug effects were reported during the study.

The initial characteristics of the study population is described in Table 1.These two randomly selected groups were well matched as to body mass index (BMI), diabetes or hyperlipidemia, which was observed in the initial diagnosis.

The liver function tests and the ultrasonographic examination were per formed regularly. There was no statistically significant change in the AST and ALP serum levels during these three months; the result was the same for both groups. While NAC can significantly decrease the ALT level, no similar changes were noted in patients receiving Vitamin C. Table 2 describes the effects of NAC, including details observed on initial diagnosis and in repeated evaluations at one-month intervals.

**Figure 1:**
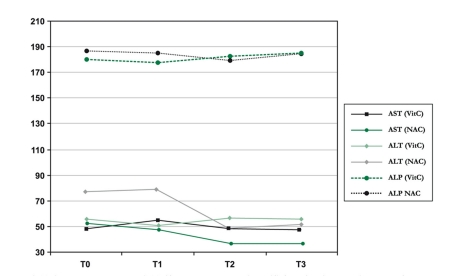
Estimated marginal means of serum liver tests in the patients receiving the grape seed extract during three months.

Fig 1. 1 shows the estimated marginal means, with a significant decrease in ALT. This effect was independent of the initial grade of steatosis.

The size of the liver, the spleen and the portal vein were evaluated by sonography. The results are described in Table 2 as well. No significant change in the size of the liver or the portal vein was observed in both groups, while the spleen size decreased significantly in the NAC group. Moreover no significant change was observed in the grade of steatosis of the liver.

**Table 1:** The comparison between the two study groups regarding age and aspects of metabolic syndrome.

	**NAC (n=15)**	**Vitamin C (n=15)**	**P**
**Mean age** (SD)	40.1(12.4)	46.8(1.4)	0.137
**Body mass index**	30.3(5.0)	33.2(6.7)	0.201
**Diabetes mellitus no**(%)	1(6.7)	2(13.3)	0.534
**Hyperlipidemia no**(%)	2(13.3)	1(6.7)	0.534
**Hypertension **	1(6.7)	1(6.7)	1.000

**Table 2:** Serum liver tests and hemodynamic parameters in the patients with non-alcoholic fatty liver disease receiving N-acetylcysteine measured in one-month intervals as mean (SD).

	**T0**	**T1**	**T2**	**T3**	**P**
ALT^b^ (units/L)	77.0(26.6)	78.3(49.3)	49.2(21.1)	51.7(17.9)	0.014
AST^a^ (units/L)	53.3(34.1)	48.3(20.2)	37.6(11.9)	37.7(10.1)	0.162
ALP^c^ (units/L)	187.1(50.6)	185.6(36.3)	181.8(40.1)	185.5(50.2)	0.970
**Liver hemodynamic parameters**					
Liver Span (mm)	156.5(33.3)	161.3(35.6)	164.8(29.3)	157.5(32.3)	0.367
Spleen Span (mm)	121.3(16.5)	121.2(15.3)	115.3(16.1)	116.2(16.5)	0.018
Portal vein diameter (mm)	9.3(1.4)	9.6(2.0)	9.5(1.8)	10.0(1.5)	0.624
**Grade of liver steatosis**					
Normal	0	1(6.7)	1(6.7)	1(6.7)	0.207
Grade 1	10(66.7)	10(66.7)	11(73.3)	12(80.0)	
Grade 2	5(33.3)	4(26.7)	3(20.0)	2(13.3)	

^a^ AST:Aspartate transaminase.

^b^ ALT:Alanine transaminase.

^c^ ALP:Alkaline phosphatase.

## Conclusions

The current study described the effectiveness of a three-month consumption of NAC in patients with NAFLD, which resulted in a decrease in the level of ALT and in the size of the spleen, compared to those patients taking vitamin C. To our knowledge, few studies have been carried out to examine the implication of NAC on liver function in patients with NAFLD, and in particular on liver hemodynamics.

Macrovesicular steatosis in the absence of significant alcohol consumption is the main characteristic of NAFLD, as a spectrum of liver diseases. This condition may be a precursor of NASH which has been found to lead to progressive fibrosis and cirrhosis. Impaired cellular function has contributed to oxidative stress leading to cellular death and hepatic necrosis caused by free radicals [11][12]. This impaired cellular function can be traced by liver function tests, which may even be the only indicator, up until the point of serious  liver damage.

Antioxidant supplements may protect cellular structures against oxidative stress. Such studies have been carried out by investigating the therapeutic interventions for NAFLD and NASH against oxidative stresses, but so far there has been no consensus about the therapeutic benefits [13]. The present study evaluates the therapeutic effect of NAC on patients with NAFLD compared to Vitamin C, as well as, for the first time, on hemodynamic parameters.

The ability of NAC to block the propagation of lipid peroxidation is reflected in the prevention of the onset of NAFLD by means of oral administration of S-nitroso-N-acetylcysteine in rats [14] and in the improvement of liver histology in rats with NASH after NAC treatment [9] However, the dietary addition of NAC is reported to have the same effect as diet alone and can improve the liver histopathology of NASH [15].

The clinical features of NASH and NAFLD are very similar, and there is no non-invasive tool for a definite diagnosis. We have tried to compensate for the nonattendance for a liver biopsy (for ethical reasons) by selecting patients with NAFLD who had elevated liver enzymes in order to obtain a sample more similar to that found in patients with NASH. Such a condition is believed to have a clinically significant risk of developing end-stage liver disease [16].

NAC can only decrease the serum level of ALT. The relationship between ALT and NAFLD has not been completely explained, but studies support a higher level of ALT as not only a consequence but also a predictor of the development of NASH in these patients [17]. Thus decreasing the level of ALT may be the best effect that it is possible to achieve.

The present study has evaluated the effects of antioxidants on ultrasonographic features of liver function for the first time. Fatty change in the liver means enlargement of the hepatocytes containing fat droplets. This slows down the blood flow throughout the hepatic sinusoids and may result in the development of portal hypertension. A correlation has been noted between the degree of fatty infiltration in the liver, and the size of spleen [18]. The current study suggests a decrease in the size of the spleen in NAFLD patients receiving NAC, which may reflect a reduction in fatty infiltration. These effects of NAC can be evaluated in detail by reviewing liver specimens after the intervention, and may be improved after a longer duration.

In conclusion, a three-month supplement of NAC can alter ALT and the size of the spleen in patients with NAFLD, which is compatible with the improvement of fatty infiltration as has been shown in earlier studies.

## References

[1] Angulo P (2002). Nonalcoholic fatty liver disease. N Engl J Med.

[2] Angulo P, Keach JC, Batts KP, Lindor KD (1999). Independent predictors of liver fibrosis in patients with nonalcoholic steatohepatitis. Hepatology.

[3] Harrison SA, Torgerson S, Hayashi P, Ward J, Schenker S (2003). Vitamin E and vitamin C treatment improves fibrosis in patients with nonalcoholic steatohepatitis. Am J Gastroenterol.

[4] Oliveira CP, Gayotto LC, Tatai C, Della Nina BI, Lima ES, Abdalla DS, Lopasso FP, Laurindo FR, Carrilho FJ (2003). Vitamin C and vitamin E in prevention of Nonalcoholic Fatty Liver Disease (NAFLD) in choline deficient diet fed rats. Nutr J.

[5] Attri S, Rana SV, Vaiphie K, Katyal R, Sodhi CP, Kanwar S, Singh K (2001). Protective effect of N-acetylcysteine in isoniazid induced hepatic injury in growing rats. Indian J Exp Biol.

[6] Caglikulekci M, Dirlik M, Pata C, Plasse M, Tamer L, Ogetman Z, Ercan B (2006). Effect of N-acetylcysteine on blood and tissue lipid peroxidation in lipopolysaccharide-induced obstructive jaundice. J Invest Surg.

[7] Huang H, Yin R, Zhu J, Feng X, Wang C, Sheng Y, Dong G, Li D, Jing H (2007). Protective effects of melatonin and N-acetylcysteine on hepatic injury in a rat cardiopulmonary bypass model. J Surg Res.

[8] Terneus MV, Kiningham KK, Carpenter AB, Sullivan SB, Valentovic MA (2007). Comparison of S-Adenosyl-L-methionine and N-acetylcysteine protective effects on acetaminophen hepatic toxicity. J Pharmacol Exp Ther.

[9] Thong-Ngam D, Samuhasaneeto S, Kulaputana O, Klaikeaw N (2007). N-acetylcysteine attenuates oxidative stress and liver pathology in rats with non-alcoholic steatohepatitis. World J Gastroenterol.

[10] de Oliveira CP, Stefano JT, de Siqueira ER, Silva LS, de Campos Mazo DF, Lima VM, Furuya CK, Mello ES, Souza FG, Rabello F, Santos TE, Nogueira MA, Caldwell SH, Alves VA, Carrilho FJ (2008). Combination of N-acetylcysteine and metformin improves histological steatosis and fibrosis in patients with non-alcoholic steatohepatitis. Hepatol Res.

[11] Chitturi S, Farrell GC (2001). Etiopathogenesis of nonalcoholic steatohepatitis. Semin Liver Dis.

[12] Starkel P, Sempoux C, Leclercq I (2003). Oxidative stress, KLF6 and transforming growth factor-beta up-regulation differentiate non-alcoholic steatohepatitis progressing to fibrosis from uncomplicated steatosis in rats. J Hepatol.

[13] Lirussi F, Azzalini L, Orando S, Orlando R, Angelico F (2007). Antioxidant supplements for non-alcoholic fatty liver disease and/or steatohepatitis. Cochrane Database Syst Rev.

[14] de Oliveira CP, Simplicio FI, de Lima VM, Yuahasi K, Lopasso FP, Alves VA, Abdalla DS, Carrilho FJ, Laurindo FR, de Oliveira MG (2006). Oral administration of S-nitroso-N-acetylcysteine prevents the onset of non alcoholic fatty liver disease in rats. World J Gastroenterol.

[15] Samuhasaneeto S, Thong-Ngam D, Kulaputana O, Patumraj S, Klaikeaw N (2007). Effects of N-acetylcysteine on oxidative stress in rats with non-alcoholic steatohepatitis. J Med Assoc Thai.

[16] Ekstedt M, Franzén LE, Mathiesen UL, Thorelius L, Holmqvist M, Bodemar G, Kechagias S (2006). Long-term follow-up of patients with NAFLD and elevated liver enzymes. Hepatology.

[17] Schindhelm RK, Diamant M, Dekker JM, Tushuizen ME, Teerlink T, Heine RJ (2006). Alanine aminotransferase as a marker of non-alcoholic fatty liver disease in relation to type 2 diabetes mellitus and cardiovascular disease. Diabetes Metab Res Rev.

[18] Tsushima Y, Endo K (2000). Spleen enlargement in patients with nonalcoholic fatty liver: correlation between degree of fatty infiltration in liver and size of spleen. Dig Dis Sci.

